# *Sasa veitchii* extract protects against carbon tetrachloride-induced hepatic fibrosis in mice

**DOI:** 10.1186/s12199-018-0739-7

**Published:** 2018-10-15

**Authors:** Hiroki Yoshioka, Tsunemasa Nonogaki, Shiori Fukaya, Yoshimi Ichimaru, Akito Nagatsu, Masae Yoshikawa, Hirohisa Fujii, Makoto Nakao

**Affiliations:** College of Pharmacy, Kinjo Gakuin University, 2-1723 Omori, Moriyamaku, Nagoya, Aichi 463-8521 Japan

**Keywords:** Carbon tetrachloride, Liver fibrosis, *Sasa veitchii*, Mitogen-activated protein kinase, Nuclear factor kappa B

## Abstract

**Background:**

The current study aimed to investigate the hepatoprotective effects of *Sasa veitchii* extract (SE) on carbon tetrachloride (CCl_4_)-induced liver fibrosis in mice.

**Methods:**

Male C57BL/6J mice were intraperitoneally injected with CCl_4_ dissolved in olive oil (1 g/kg) twice per week for 8 weeks. SE (0.1 mL) was administered orally once per day throughout the study, and body weight was measured weekly. Seventy-two hours after the final CCl_4_ injection, mice were euthanized and plasma samples were collected. The liver and kidneys were collected and weighed.

**Results:**

CCl_4_ administration increased liver weight, decreased body weight, elevated plasma alanine aminotransferase, and aspartate aminotransferase and increased liver oxidative stress (malondialdehyde and glutathione). These increases were attenuated by SE treatment. Overexpression of tumor necrosis factor-α was also reversed following SE treatment. Furthermore, CCl_4_-induced increases in α-smooth muscle actin, a marker for hepatic fibrosis, were attenuated in mice treated with SE. Moreover, SE inhibited CCl_4_-induced nuclear translocation of hepatic nuclear factor kappa B (NF-κB) p65 and phosphorylation of mitogen-activated protein kinase (MAPK).

**Conclusion:**

These results suggested that SE prevented CCl_4_-induced hepatic fibrosis by inhibiting the MAPK and NF-κB signaling pathways.

## Background

The liver performs important functions such as lipid metabolism, protein synthesis, and circulatory detoxification [1]. Acute or chronic hepatic injury can be caused by viral infections, non-alcoholic steatohepatitis, toxins, and alcoholism [2, 3]. The pathological progression of hepatic cell damage can result in fibrosis, cirrhosis, and ultimately hepatocellular carcinoma [4]. Hepatic fibrosis, a gradually progressing chronic liver disease, is a wound-healing response of the liver to repeated injury. Following acute injury, hepatocytes regenerate and replace necrotic or apoptotic cells, a process associated with inflammatory response. Hepatic fibrosis results from excessive accumulation of scar tissue following inflammation and liver cell death that occurs in most types of chronic liver disease.

Chronic exposure to carbon tetrachloride (CCl_4_) is used to establish an experimental model of chronic liver disease, resulting in oxidative stress and hepatic injury. The mechanism of CCl_4_-induced hepatotoxicity is well characterized [5] and is mediated by radicals generated by CYP2E1, such as trichloromethyl and trichloromethyl peroxy radicals. Increased oxidative stress mediated by reactive oxygen species (ROS) following CCl_4_ exposure may play an essential role in progression of liver damage [6]. ROS induce tissue injury via lipid peroxidation and enhance hepatic fibrosis by increasing tissue levels of metalloproteinases inhibitors, resulting in increased collagen synthesis and accumulation [7]. Antioxidant compounds, such as polyphenols, are effective in the treatment of chronic hepatic damage and fibrosis [8, 9]. Polyphenols derived from plant extracts have demonstrated hepatoprotective properties [10, 11].

*Sasa veitchii*, of the family Gramineaehas, has been used in Asia as a health-promoting food and folk medicine. In Japan, its leaves are used as a food wrapping material or sushi sheet to prevent food from rotting. *S. veitchii* extract (SE) exhibits antioxidant, antitumor, antiulcer, anti-inflammatory, antimicrobial, antiviral, and anti-allergic activities [12–17]. These effects are attributed to C-glycoside flavonoids and phenolic acids, which are abundant in these extracts [18, 19]. In our previous study, we showed that SE reduced high-fat diet-induced obesity by modulating adipose differentiation and preventing hepatic steatosis in mice [20, 21]. In addition, SE confers protection against CCl_4_- and acetaminophen-induced acute hepatic injury in mice [22, 23]. Since obesity and CCl_4_- or acetaminophen-induced acute hepatotoxicity are commonly associated with inflammatory response, SE may have protective effects against other diseases with inflammatory components, such as chronic hepatitis.

To further characterize the therapeutic benefits of SE, we examined whether hepatitis induced by chronic exposure to CCl_4_ is prevented by treatment with SE.

## Methods

### Preparation of SE

Commercial, non-prescription SE was kindly provided by Sunchlon Co., Ltd. (Nagano, Japan). One milliliter of SE was made from 2.82 g of *S. veitchii* leaves according to the company data [23]. Chemical components of SE have been previously reported [22].

### Apparatus and chromatographic conditions

Three-dimensional high-performance liquid chromatography (HPLC) analysis was performed using a JASCO series (JASCO Corporation, Tokyo, Japan) system consisting of a JASCO PU-2089 Plus pump and a JASCO MD-2010 Plus photodiode array detector. Chromatographic separation was performed using a YMC-Pack ODS-AL S-5 column (5 μm, 250 mm × 4.6 mm i.d., YMC Co., Ltd., Tokyo, Japan) controlled at 40 °C (Shimadzu CTO-20 AC, Shimadzu Corporation, Kyoto, Japan). The mobile phases consisted of acetonitrile (A) and 50 mM sodium dihydrogen phosphate monohydrate (B). Gradient elution was performed at a flow rate of 0.8 mL/min with the following parameters: 0–60 min A:B (10:90, *v*/*v*) to A:B (90:10, *v*/*v*), 60–75 min A:B (90:10, *v*/*v*), and 75–80 min A:B (10:90, *v*/*v*). The injection volume was 10 μL (0.2 mg/mL). Scan data were collected from 220 to 600 nm.

### Experimental protocol

Twenty-four male C57BL/6J mice weighing 19–21 g and aged 6 weeks were purchased from CLEA Japan, Inc. (Tokyo, Japan). Mice were housed in a temperature- and humidity-controlled environment (24 ± 1 °C and 55 ± 5%, respectively) with a standard 12 h light/dark cycle (8:00/20:00) and provided with food and water ad libitum. This experiment was approved by the Institutional Animal Care and Experimentation Committee of Kinjo Gakuin University. After acclimatization to laboratory conditions for 1 week, the mice (7-week old) were randomly divided into 4 groups (6 mice each): (1) control group, (2) SE-treated group, (3) CCl_4_-treated group, and (4) SE + CCl_4_-treated group. In the CCl_4_ and SE + CCl_4_ groups, mice were intraperitoneally (i.p.) injected with CCl_4_ (1 g/kg (5 ml/kg) at 22:00 twice per week) for 8 weeks. The control and SE groups received i.p. injections of olive oil as placebo. Mice in the SE and SE + CCl_4_ groups were orally administered SE daily (0.1 mL at 10:00 every day) for the duration of the treatment course. Saline vehicle was orally administered to the control and CCl_4_ groups.

Body weight was measured weekly throughout the study. Mice were euthanized 72 h following the final CCl_4_ injection and bled by cardiocentesis to obtain plasma samples, which were stored at − 80 °C until analysis. The liver and kidneys were weighed. In addition, separate liver samples were snap frozen in liquid nitrogen and stored at − 80 °C or fixed in 15% neutral buffered formalin (pH 7.4).

### Plasma biochemical analysis

Plasma levels of alanine aminotransferase (ALT) and aspartate aminotransferase (AST) were determined using a Transaminase CII kit (Wako Pure Chemical Industries, Osaka, Japan), as previously described [24–26]. Plasma levels of tumor necrosis factor alpha (TNFα) were measured using a commercially available ELISA kit (eBioscience, San Diego, CA, USA), as previously described [22]. For relative quantification, calibration curves were prepared using standard solutions.

### Measurement of malondialdehyde and glutathione levels in the liver

Total malondialdehyde (MDA) levels in the liver were measured using a colorimetric microplate assay kit for thiobarbituric acid reactive substances (Oxford Biochemical Research, Oxford, MI, USA), as previously described [24]. Glutathione (GSH) levels in the liver were examined using a GSSG/GSH quantification kit (Dojindo Laboratories, Kumamoto, Japan), as previously described [22].

### Histopathological analysis

A portion of the left lobe of the liver from each animal was perfused with 15% phosphate-buffered neutral formalin (pH 7.4), dehydrated, and embedded in paraffin. Embedded tissues were sliced into 4-μm thick sections and stained with hematoxylin and eosin (H&E), as previously described [27, 28]. Liver fibrosis was quantified by using Masson’s trichrome (MT) staining according to the manufacturer’s protocol (ScyTek Laboratories, Inc., Logan, UT, USA). Necrosis area and aniline blue-positive area were calculated using ImageJ software (NIH). For immunohistochemistry staining, paraffin-embedded sections were deparaffinized and rehydrated in a graded ethanol series. Following antigen retrieval using proteinase K (Wako Pure chemical) and blocking of endogenous peroxidase using hydrogen peroxide (Wako Pure chemical), sections were incubated with rat anti-p65 monoclonal antibody (Santa Cruz, CA, USA) (1:160 dilution) as a primary antibody at 4 °C overnight (16 h). The sections were then incubated with a secondary antibody, anti-mouse IgG-FITC (MBL, Aichi, Japan) (1:160 dilution). In addition, sections were counterstained with 4′,6-diamidino-2-phenylindole (DAPI) for nuclear staining.

### Western blot analysis

Liver sections (80 mg) were homogenized in 720 μL ice-cold phosphate-buffered saline containing a phosphatase inhibitor and protease inhibitor (Nacalai Tesque, Kyoto, Japan) and centrifuged at 20,000 × *g* for 15 min at 4 °C. The supernatant from each sample was collected, and protein was extracted using a BCA protein kit (Nacalai Tesque). Protein samples (30 μg) were subjected to gradient sodium dodecyl sulfate-polyacrylamide gel electrophoresis (BioRad, Hercules, CA, USA) and transferred to a polyvinylidene difluoride membrane using Trans-Blot Turbo Transfer System (BioRad). Next, the membrane was incubated with mouse anti-α-smooth muscle actin (anti-αSMA) monoclonal antibody (Santa Cruz), mouse anti-β-actin monoclonal antibody (MBL, Aichi, Japan), rabbit anti-c-Jun N-terminal kinase (JNK) polyclonal antibody, rabbit anti-phospho-JNK monoclonal antibody, rabbit-anti extracellular signal-regulated kinase (ERK) 1/2 monoclonal antibody, rabbit anti-phospho-ERK1/2 monoclonal antibody, rabbit-anti p38 monoclonal antibody, rabbit anti-phospho-p38 monoclonal antibody, peroxidase-conjugated anti-rabbit IgG (Cell Signaling Technology, Beverly, MA, USA), and peroxidase-conjugated anti-mouse IgG (BioRad) using previously described conditions [29]. Immuno-reactive bands were visualized with an ECL system (BioRad). Band intensity was measured using ImageJ software (NIH).

### Statistical analysis

Multiple comparisons were performed using one-way analysis of variance (ANOVA) with Tukey’s test. All statistical analyses were performed using SPSS Statistics for Windows software (version 24.0; IBM Corp., Armonk, NY, USA). Differences were considered statistically significant at *p* values < 0.05.

## Results

### Analysis of SE by 3D-HPLC

**Fig. 1:**
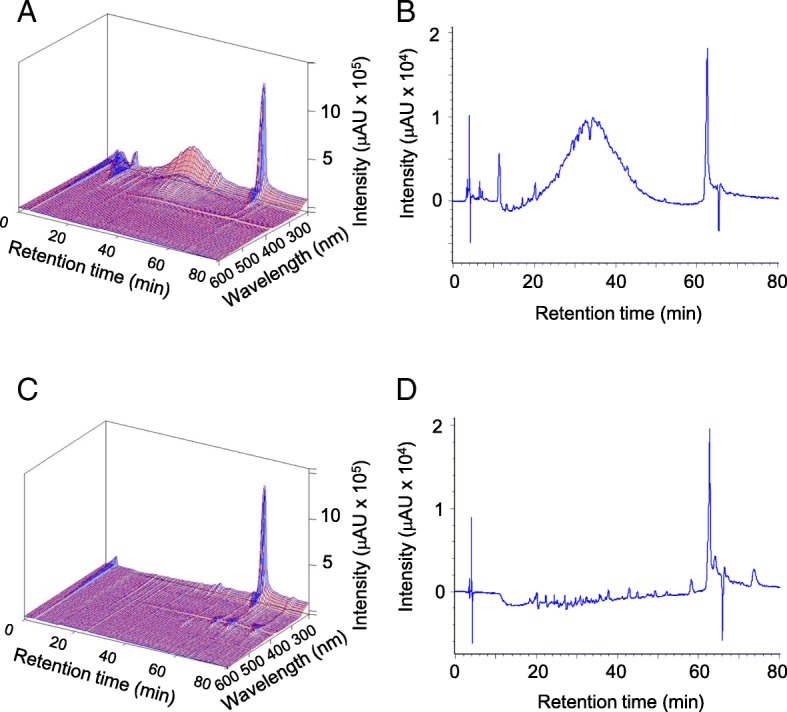
3D-HPLC of *S. veitchii* leaf extract (SE) and sodium copper chlorophyllin (SCC). Panels **a** and **c** indicate the 3D-HPLC fingerprint of SE and SCC, respectively. Panels **b** and **d** indicate the 2D-HPLC fingerprint at 254 nm of SE and SCC, respectively. Separation was achieved using a YMC-Pack ODS-AL S-5 column. The mobile phases consisted of CH_3_CN (**a**) and 50 mM NaH_2_PO_4_ aq. (**b**); linear gradient elution was performed at a flow rate of 0.8 mL/min for 60 min A:B = 10:90 (*v*/*v*) to 90:10 (*v*/*v*) and followed by isocratic condition of B:A = 90:10 (*v*/*v*) for 15 min, then B:A = 10:90 (*v*/*v*) for 5 min. The column oven temperature was held at 40 °C. Scan data were collected across the range of 220–600 nm.

Typical chromatograms of SE and sodium copper chlorophyllin (SCC), the active ingredient of SE, are shown in Fig. 1. SCC eluted at a retention time of 63 min (Fig. 1c, d). However, various components in addition to SCC were present in SE (Fig. 1a, b). The broad peak in the SE chromatogram absorbed at 254 nm, suggesting the presence of organic compounds (Fig. 1b).

### Body weight change and organ weight

**Fig. 2:**
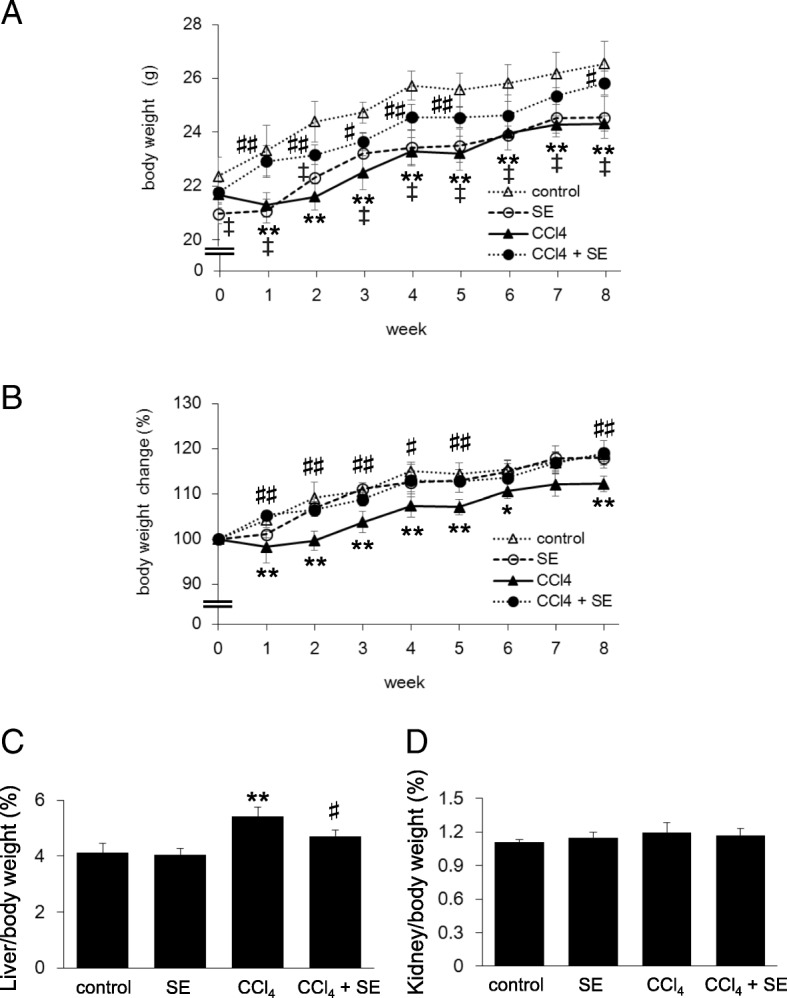
Effect of SE on body weight change resulting from CCl_4_-induced hepatotoxicity. Mice were given CCl_4_ dissolved in olive oil intraperitoneally twice per week for 8 weeks. The control and SE groups received olive oil. At all periods of the experiment, mice were treated daily with SE or saline. Body weights were measured weekly throughout the study. Body weight and body weight change were calculated from the beginning of the study. Animals were euthanized 72 h after the final CCl_4_ injection. Panels **a**, **b**, **c**, and **d** indicate body weight, body weight change ratio, liver weight ratio, and kidney weight ratio, respectively. The data represent the mean ± SD of 6 mice per group; ^‡^*p* < 0.01 (control versus SE group), **p < 0.01 (control versus CCl_4_ group), and ^#^*p* < 0.05 and ^##^*p* < 0.01 (CCl_4_ versus CCl_4_ + SE group).

We monitored change in body weight each week for 8 weeks. Throughout the experiment (from 1 week to 8 week), exposure to CCl_4_ significantly decreased body weight compared to control (*p* < 0.01) (Fig. 2a). Not only absolute body weights but also body weight change was observed the same tendency (Fig. 2b)*.* In contrast, body weight and body weight change in the CCl_4_ + SE group was significantly higher than that in the CCl_4_ group at 1, 2, 3, 4, 5, and 8 weeks. Body weight in the SE group was also significantly lower than that in the control group throughout the experiment including at 0 week. Since our previous study indicated that SE treatment for 2 weeks has no significant change on body weight in normal mice [21], lower starting body weight of mice in the SE group may be responsible for this difference. Therefore, we showed body weight ratios in Fig. 2b. Body weight in the control and SE groups continued to increase throughout the experiment, with overall changes of 118.7 ± 1.5 and 117.9 ± 2.3%, respectively. These data suggest that SE administration for 8 weeks did not significantly affect body weight change. In addition, the body weight changes in the CCl_4_ and CCl_4_ + SE groups correlated with body weight. These data indicated that SE may counteract body weight decreases induced by CCl_4_.

To examine the effects of SE and chronic exposure to CCl_4_ on organ weight, we measured the weights of the liver and kidney samples*.* The liver/body weight ratio was significantly higher (*p < 0.01*) in mice injected with CCl_4_ than control levels (Fig. 2c). In contrast, SE treatment alleviated CCl_4_-induced elevation of liver/body weight ratio (*p < 0.05*). Moreover, the kidney/body weight ratios of the four groups were not different (Fig. 2d). These data indicated that SE prevented CCl_4_-induced decreases in body weight and increases in relative liver weight.

### Plasma biochemical parameters and oxidative stress

**Fig. 3:**
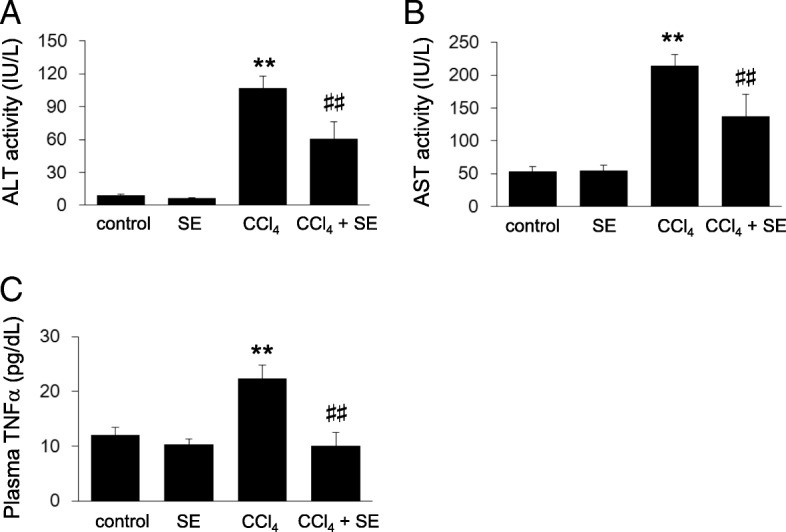
Effect of SE on levels of hepatic injury markers and inflammatory cytokines. Animals treated as described in Fig. 2 were euthanized 72 h post intraperitoneal injection and plasma levels of hepatic injury markers, and inflammatory cytokines were determined. Panels **a**, **b**, and **c** indicate ALT, AST, and TNFα, respectively. Data are plotted as mean ± SD for groups of 6 mice each. ** indicates *p < 0.01* versus control group, and ^##^ indicates *p < 0.01* versus CCl_4_ group.

As shown in Fig. 3, we determined plasma levels of several biochemical markers. Plasma levels of hepatic injury markers, ALT and AST, significantly increased following chronic CCl_4_ exposure, and this increase was attenuated by SE treatment (Fig. 3a b, respectively).

In addition, we measured plasma TNFα, a marker of chronic hepatitis and inflammation [30, 31]. CCl_4_ administration for 8 weeks potentiated the levels of plasma TNFα, and SE treatment significantly attenuated this effect (Fig. 3c).

### Oxidative stress

**Fig. 4:**
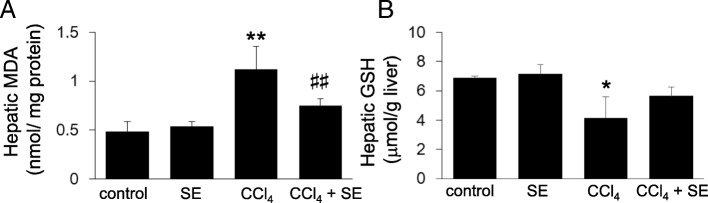
Effect of SE on the levels of hepatic MDA and GSH levels. Animals treated as described in Fig. 2 were euthanized 72 h post intraperitoneal injection, and the livers were harvested at necropsy. Liver specimens were assessed for MDA levels (**a**) and GSH levels (**b**). Data are plotted as mean ± SD for groups of 6 mice each. * indicates *p < 0.05* versus control group*,* ** indicates *p < 0.01* versus control group, and ^##^ indicates *p < 0.01* versus CCl_4_ group.

MDA is a marker of lipid peroxidation [32]. Hepatic MDA levels in the CCl_4_ group were significantly higher (*p* < 0.01) than those in the normal control group (Fig. 4a). The SE treatment group had significantly lower (*p* < 0.01) MDA levels than the CCl_4_ treatment group. In contrast, GSH, a major intracellular antioxidant [33], was significantly lower in the CCl_4_ treatment group than in the normal group (Fig. 4b). Although not significant (*p = 0.096*), treatment with SE attenuated CCl_4_-induced reduction of GSH levels in the liver.

### Histopathology

**Fig. 5:**
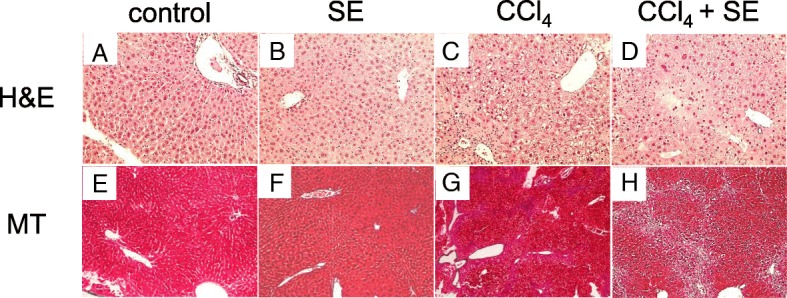
SE treatment protects animals from repeated CCl_4_-induced hepatotoxicity, as assessed by H&E staining and MT staining. Animals treated as described in Fig. 2 were euthanized at 72 h post intraperitoneal injection, and the livers were harvested at necropsy. Liver specimens were fixed and processed by standard methods, and sections were stained with H&E and MT. Data in **a**–**d** indicate H&E stain and **e**–**h** indicate MT stain, respectively. Necrotic area and aniline blue-positive area were calculated using ImageJ software.

Along with measurement of plasma biochemical parameters and hepatic oxidative stress, we conducted histopathological studies of liver tissues (Fig. 5)*.* Liver sections of control (Fig. 5a) and SE (Fig. 5b) groups stained with H&E showed normal hepatic architecture. In contrast, the livers of CCl_4_-administered mice showed massive necrosis (62.1%) accompanied with microvesicular steatosis and mononuclear cell infiltration (Fig. 5c). Compared to the CCl_4_ group, hepatic injury was attenuated in the group treated with SE (17.4% necrotic) (Fig. 5d).

In addition, we observed collagen deposition in the tissues using MT staining. The livers from the control (Fig. 5e) and SE groups (Fig. 5f) showed normal hepatic architecture, with collagen fibrils visible only in the walls of adjacent blood vessels. In contrast, mice administered CCl_4_ for 8 weeks showed fibrotic scars within hepatic lesions, predominantly in the periportal areas, with early fibrotic septa developed as thin fibrotic strands (aniline blue-positive area was 4.3%) (Fig. 5g). In mice treated with SE, the area of collagen deposition was lower than that in the CCl_4_ group (aniline blue-positive area was 1.9%) (Fig. 5h).

### αSMA expression

**Fig. 6:**
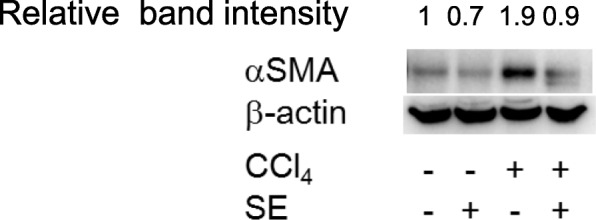
Effect of SE on levels of hepatic αSMA levels. Animals treated as described in Fig. 2 were euthanized at 72 h post intraperitoneal injection, and liver tissues were isolated. Proteins were isolated, and western blotting was performed. β-actin was used as the internal control. Band intensity was measured using ImageJ software.

Expression levels of αSMA, a marker for hepatic fibrosis [34], were determined by immunoblotting (Fig. 6). Long-term CCl_4_ administration induced expression of αSMA, whereas SE treatment attenuated the CCl_4_-induced increase in hepatic αSMA expression. These results were consistent with histopathology results. In addition, similar results were observed using immunohistochemistry analyses (data not shown).

### Localization of p65

**Fig. 7:**
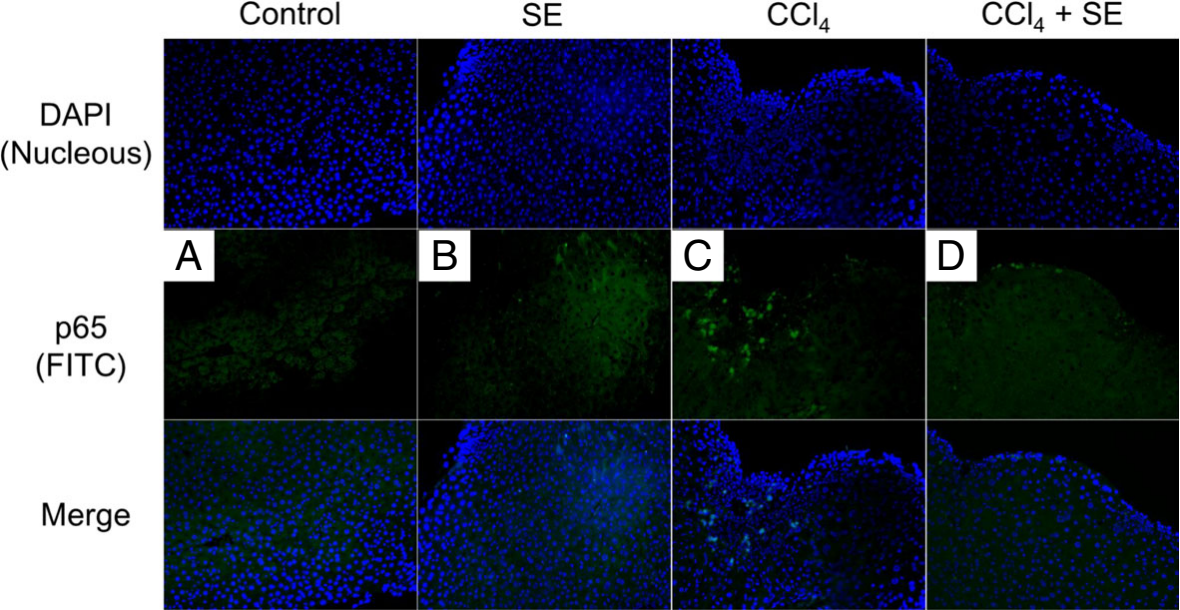
Effect of treatment with SE on liver p65 fluorescent immunostaining. Animals treated as described in Fig. 2 were euthanized at 72 h post intraperitoneal injection, and the livers were harvested at necropsy. Liver specimens were removed, fixed, and processed by standard methods. Nuclei were counterstained with DAPI (blue). Expression and localization of p65 (green) were analyzed using rabbit anti-p65 monoclonal antibody and anti-mouse IgG-FITC. Panel in **a**-**d** indicate control, SE, CCl_4_, and CCl_4_ + SE, respectively

Next, we evaluated the effect of SE on CCl_4_-induced p65 activation using immunohistochemistry (Fig. 7). A large proportion of p65 in the livers of the control (Fig. 7a) and SE groups (Fig. 7b) was localized in the cytoplasm. In contrast, the level of nuclear p65 significantly increased in the livers of mice injected with CCl_4_ (Fig. 7c). SE treatment inhibited the nuclear translocation of p65 (Fig. 7d).

### MAPK signaling pathway

**Fig. 8:**
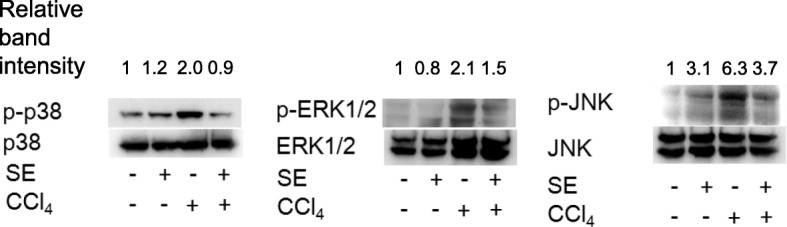
Effect of SE on phosphorylation of JNK, p38, and ERK1/2 in the liver. Animals treated as described in Fig. 2 were euthanized at 72 h post intraperitoneal injection, and the liver tissues were isolated. Proteins were isolated and western blotting was performed. The data indicated increased hepatic phosphorylation of JNK, p38, and ERK1/2, demonstrating ERK1/2 activation of each MAPK. Total JNK, p38, and ERK1/2 levels were measured as internal controls. Band intensity was measured using ImageJ software.

We determined the effects of SE on CCl_4_-induced hepatic mitogen-activated protein kinase (MAPK) signaling (Fig. 8). We confirmed p38, ERK, and JNK phosphorylation following 8 weeks of CCl_4_ administration. Treatment with SE attenuated CCl_4_-induced MAPK (p38, ERK, and JNK) phosphorylation. These results suggest that SE may prevent fibrogenesis by downregulating the MAPK signaling pathway.

## Discussion

Hepatic fibrosis is a consequence of chronic liver damage associated with an increased morbidity rate and is considered a worldwide health risk [2, 35]. Therefore, prevention of chronic liver fibrosis is important to improve our quality of life. CCl_4_ is the most commonly used toxin for inducing hepatic fibrosis in experimental models owing to the similarity of CCl_4_-induced hepatic fibrosis to hepatic fibrosis in humans. Repeated exposure to CCl_4_ is known to enhance fibrogenesis in hepatic tissues of mice [36]. CCl_4_ is converted to trichloromethyl free radicals in the liver by CYP2E1, resulting in lipid peroxidation and liver injury [5]. In the current study, we established a hepatic fibrosis model through repeated exposure to CCl_4_ and evaluated the protective effects of SE against hepatic fibrosis.

Oxidative stress is a major cause of hepatic fibrosis progression in experimental models [37]. Lipid peroxidation, a marker of oxidative damage, is associated with a wide range of diseases [38]. Moreover, oxidative stress promotes collagen synthesis by activated hepatic stellate cells (HSC) [39]. Increased levels of MDA, a product of lipid peroxidation, in hepatic tissues reflect oxidative stress in hepatic cells [32]. Therefore, many antioxidants have been investigated as preventive and therapeutic agents against CCl_4_-induced hepatic fibrosis [40–43]. The present study showed that SE treatment decreased CCl_4_-induced increases in ALT and AST levels, collagen deposition, and MDA production. These findings suggested that SE attenuated hepatic fibrosis through antioxidant activity, consistent with our previous study that reported the antioxidant property of SE [23].

HSCs are major fibrotic precursor cells that transdifferentiate to the extracellular matrix, producing myofibroblasts [34]. The present study showed that αSMA, a marker of the initiation phase of HSC activation, was elevated in the liver and was accompanied by bridging fibrosis. In addition, CCl_4_-induced increases in αSMA were attenuated in the livers of mice treated with SE. These results suggested that SE inhibited HSC activation during the pathogenesis of CCl_4_-induced liver fibrosis. Increasing evidence indicates that inflammatory cytokines, such as TNFα, are critical to HSC activation in the pathogenesis of liver fibrosis [44]. Moreover, the relationship between NF-κB and inflammatory response is well characterized [45, 46]. NF-κB is sequestered in the cytoplasm by binding to IκB. In response to stress or pro-inflammatory stimuli, NF-κB translocates to the nucleus through translational inhibition of IκB [47]. Our present study showed that plasma levels of TNFα were significantly elevated in mice administered CCl_4_. CCl_4_-induced hepatic NF-κB nuclear translocation was inhibited in mice administered SE, indicating that the protective effect of SE was associated with decreased TNFα production and subsequent improvement of liver histopathology. These results suggested that SE suppressed CCl_4_-induced HSC activation by inhibiting NF-κB activation and subsequent inflammatory response.

The MAPK family regulates cellular proliferation in response to cellular stresses and inflammation [48, 49]. Three distinct MAPK pathways have been reported in mammalian cells: the c-Jun N-terminal kinase (JNK) pathway, extracellular signal-regulated kinase (ERK) pathway, and p38 MAPK pathway. These three pathways are associated with HSC activation in the pathogenesis of hepatic fibrosis [50, 51]. Moreover, CCl_4_ activates MAPK signaling [35, 42]. The present study showed that SE treatment attenuated CCl_4_-induced phosphorylation of hepatic JNK, ERK1/2, and p38. These results suggest that SE inhibited CCl_4_-induced hepatic oxidative stress, inflammatory response, and HSC activation by attenuating the activation of MAPK signaling.

Most studies, including ours, utilized an extract obtained from *S. veitchii* leaves, which are rich in bioactive compounds. The SE used in this study contained an abundance of SCC (250 μg/mL) [23], which exerts anti-inflammatory and antioxidant effects [52, 53]. Prior to this study, the active ingredient of SE had not been characterized. As such, we performed HPLC analysis to confirm the components of SE. A broad peak and a sharp peak were observed. The sharp peak corresponded to SCC contained in SE. Because the activities of SE are different from those of SCC, we speculated that the active ingredient of SE described in the present study is represented by the broad peak. Several antioxidant and anti-inflammatory compounds, such as polyphenols and flavonoids, have also been identified in *S. veitchii* [18, 54], indicating that these components may mediate the anti-fibrotic effects of SE. Since our present study was conducted using crude SE extracts, further investigation is needed to elucidate the active components of SE.

## Conclusion

**Fig. 9:**
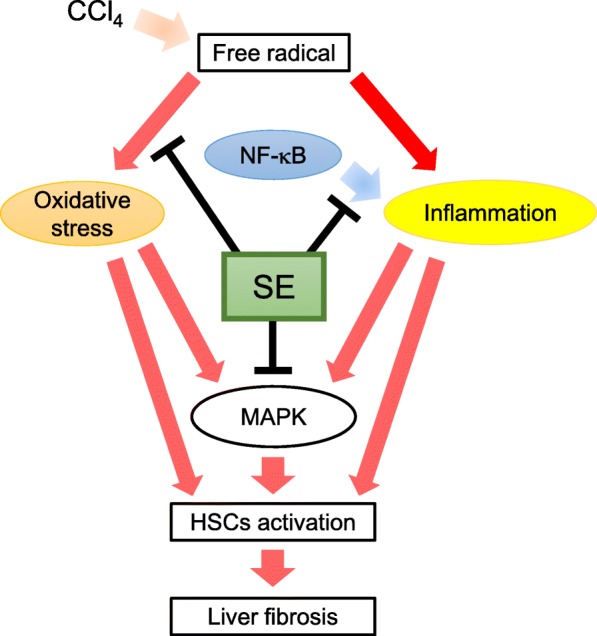
Proposed protective mechanism of SE on CCl_4_-induced liver fibrosis

The present study showed that SE treatment prevented CCl_4_-induced hepatic injury. Moreover, SE attenuated CCl_4_-induced inflammatory response and subsequent HSC activation, hepatic NF-κB translocation, and MAPK signaling (Fig. 9). Hence, SE may have potential preventive/therapeutic potential for the treatment of hepatic fibrosis. Although repeated administration of CCl_4_ was mimic hepatic fibrosis model, our present investigation is valuable to contribute to self-medication options against chronic hepatic injury and disease.

## Abbreviations

ALT: Alanine aminotransferase
AST: Aspartate aminotransferase
CCl_4_: Carbon tetrachloride
DAPI: 4′,6-Diamidino-2-phenylindole
ERK: Extracellular signal-regulated kinase
GSH: Glutathione
H&E: Hematoxylin and eosin
HPLC: High-performance liquid chromatography
HSC: Hepatic stellate cells
i.p.: Intraperitoneally
JNK: c-Jun N-terminal kinase
MAPK: Mitogen activated protein kinases
MDA: Malondialdehyde
MT: Masson’s trichrome
NF-κB: Nuclear factor kappa B
ROS: Reactive oxygen species
SCC: Sodium copper chlorophyllin
SE: *Sasa veitchii* extract
TNFα: Tumor necrosis factor alpha
αSMA: α-Smooth muscle actin
